# Family-supportive manager and perceived pregnancy discrimination: psychological contract breach and trust as mediators of remote working mothers’ turnover intentions

**DOI:** 10.3389/fpsyg.2026.1953215

**Published:** 2026-10-13

**Authors:** Aviv Kidron, Neta Cohen, Michal Gross

**Affiliations:** 1 Department of M.A. in Organizational Development and Consulting, The Max Stern Yezreel Valley College, Yezreel Valley, Israel; 2 Department of Economics and Management and Department of Human Services, The Max Stern Yezreel Valley College, Yezreel Valley, Israel; 3 Department of Human Services and Department of Social Work, The Max Stern Yezreel Valley College, Yezreel Valley, Israel

**Keywords:** family-supportive manager, hybrid work, organizational trust, perceived pregnancy discrimination, pregnant employees, psychological contract breach, remote work, turnover intentions

## Abstract

**Introduction:**

The potential impact of remote work on the continuity of women’s careers may be contingent upon managerial attitudes and organizational norms. However, research on pregnant employees and those on post maternity-leave who work remotely has been limited. The present study proposes an integrative model examining the indirect associations of family-supportive manager and perceived pregnancy discrimination with turnover intentions through two mediators: psychological contract breach and organizational trust.

**Method:**

We conducted an online survey in two waves, with 406 women either pregnant or on post maternity-leave who worked remotely or in a hybrid arrangement.

**Findings:**

The results show that psychological contract breach mediated the relationship between family-supportive manager and organizational trust (indirect = 0.08, *p* < 0.05). Similarly, psychological contract breach mediated the relationship between perceived pregnancy discrimination and organizational trust (indirect = −0.19, *p* < 0.001). Organizational trust mediated the effect of psychological contract breach on turnover intentions (indirect = 0.11, *p* < 0.01), although the mediation was complementary to the direct effect rather than complete. A complete indirect effect of a family-supportive manager on turnover intentions was found through the combined mediation of psychological contract breach and organizational trust (indirect = −0.07, *p* < 0.01; indirect = −0.08, *p* < 0.01; indirect = −0.02, *p* < 0.01; respectively) and of perceived pregnancy discrimination on turnover intentions through the combined mediation of psychological contract breach and organizational trust (indirect = −0.05, *p* < 0.01; indirect = 0.16, *p* < 0.001, respectively).

**Discussion:**

Theoretically, this study generates new theoretical insights by synthesizing the theories of gendered organizations and work-family boundary. The study offers preliminary insights into the relationship between family-supportive manager and perceived pregnancy discrimination with the psychological contract breach that may result. The study extends previous research on turnover intentions by examining indirect associations linking family-supportive manager and perceived pregnancy discrimination to turnover intentions through psychological contract breach and organizational trust. Practically, this study suggests that family-friendly policies may be effective if, in parallel, organizations redefine their work relations to create a supportive work environment for pregnant and post maternity-leave employees.

## 1 Introduction

Remote working has received a lot of attention in recent years (Barhate and Hirudayaraj, 2021; Reifenscheid and Möhring, 2025) and has become one of the main demands of employees from their employers. Working remotely allows employees to better manage the boundaries between work and other personal activities (Gashi et al., 2021), and it is particularly attractive to women seeking independence and flexibility in balancing their careers with family responsibilities (Gatto, 2020). Remote or hybrid work can be a strategy to plug the “leaky pipeline” of pregnant and post-maternity leave employees, thereby facilitating the maintenance of career continuity (Agarwal and Nelson, 2023; Hashmi et al., 2021). As remote work arrangements continue to expand, this study proposes a model examining the indirect relationships of family-supportive manager behavior and perceived pregnancy discrimination with turnover intentions through two mediators, psychological contract breach and organizational trust, among pregnant employees and employees who have returned from maternity leave.

This issue is especially crucial when we consider the employment context of women in the Israeli labor market. The employment rate of women in Israel is high, at 75.6% (Weiss, 2023). Concurrently, in 2023, the fertility rate in Israel was 3 children per woman, the highest in the OECD, and nearly twice the OECD average of 1.58. Maternity leave, contingent on the employee’s social security payments, ranges between 8 and 15 weeks in Israel, whereas the average maternity leave in OECD countries is 18 weeks. The Equal Opportunities at Work Commission report (2023) indicates a notable increase in claims of pregnancy discrimination (17%) compared to the previous year (13%). Moreover, 42% of the women’s complaints had to do with discrimination based on family responsibilities, such as expanding their families or caring for them. The Israel Women’s Network reports that one-third of inquiries concern violations of women’s employment rights during fertility treatments, pregnancy, and the postpartum period (Maron and Hirsch, 2025). These violations undermine women’s return to work and ultimately lead to job loss. Navon (2026) found that one in six employed mothers experienced unfair treatment during pregnancy, showing that pregnancy-related workplace discrimination exists across various sectors and occupations.

Regarding remote work, a survey conducted by the Israel Democracy Institute (2023) found that 68% of women stated that they would like to work more days from home, compared to 52% of men, especially female employees with children under the age of 12. These troubling facts motivate us to examine the factors that influence the intentions of pregnant and post-maternity leave employees to quit their jobs.

Therefore, the present study makes three contributions: First, the study provides new theoretical insights by synthesizing the theory of gendered organizations (Acker, 1990) and the theory of the work-family boundary (Clark, 2000). Recently, it has been argued that Acker’s theory needs to be updated because of the changing nature of the world of work (Bates, 2022). This study builds upon Acker's (1990) theory of gendered organizations, identifying new mechanisms that could perpetuate gender inequality in remote and hybrid working environments. Organizations can influence and change the boundaries of work domains without following up with corresponding changes in workplace policies, cultures, or attitudes (Clark, 2000) that allow women to benefit from remote work opportunities (Barhate and Hirudayaraj, 2021). Flexible work arrangements have been shown to reduce turnover (Hashmi et al., 2021), but according to work-family boundary theory, remote work may carry a stigma that undermines women’s motivation to be effective boundary crossers (Barhate and Hirudayaraj, 2021). Yet, there has been limited research on gender in remote work (Touzet, 2023).

Second, this study responds to Hyunkang and Gordon's (2024) call for an investigation into the potential mechanisms underlying turnover intentions, with a particular focus on the attitudes of pregnant and post-maternity employees. Generally, a global study in 37 countries found that women who plan to have children are more likely than men to intend to quit their jobs, which is negatively associated with career ambition (Olsson et al., 2023). The direct relationship between psychological contract breach and organizational trust has already been studied (e.g., Kidron and Gedalya, 2026), as has the relationship between trust and turnover intentions (Pathardikar et al., 2025; Zhao et al., 2022). However, research on the psychological contract breach of post-maternity leave employees is scarce (Varma and Sivarajan, 2024), as is research on their trust in organizations. This study offers a new perspective by proposing that family-supportive management and perceived pregnancy discrimination are indirectly associated with turnover intentions through psychological contract breach and organizational trust (Figure 1). To date, these mediators have been examined mostly separately.

**Figure 1:**
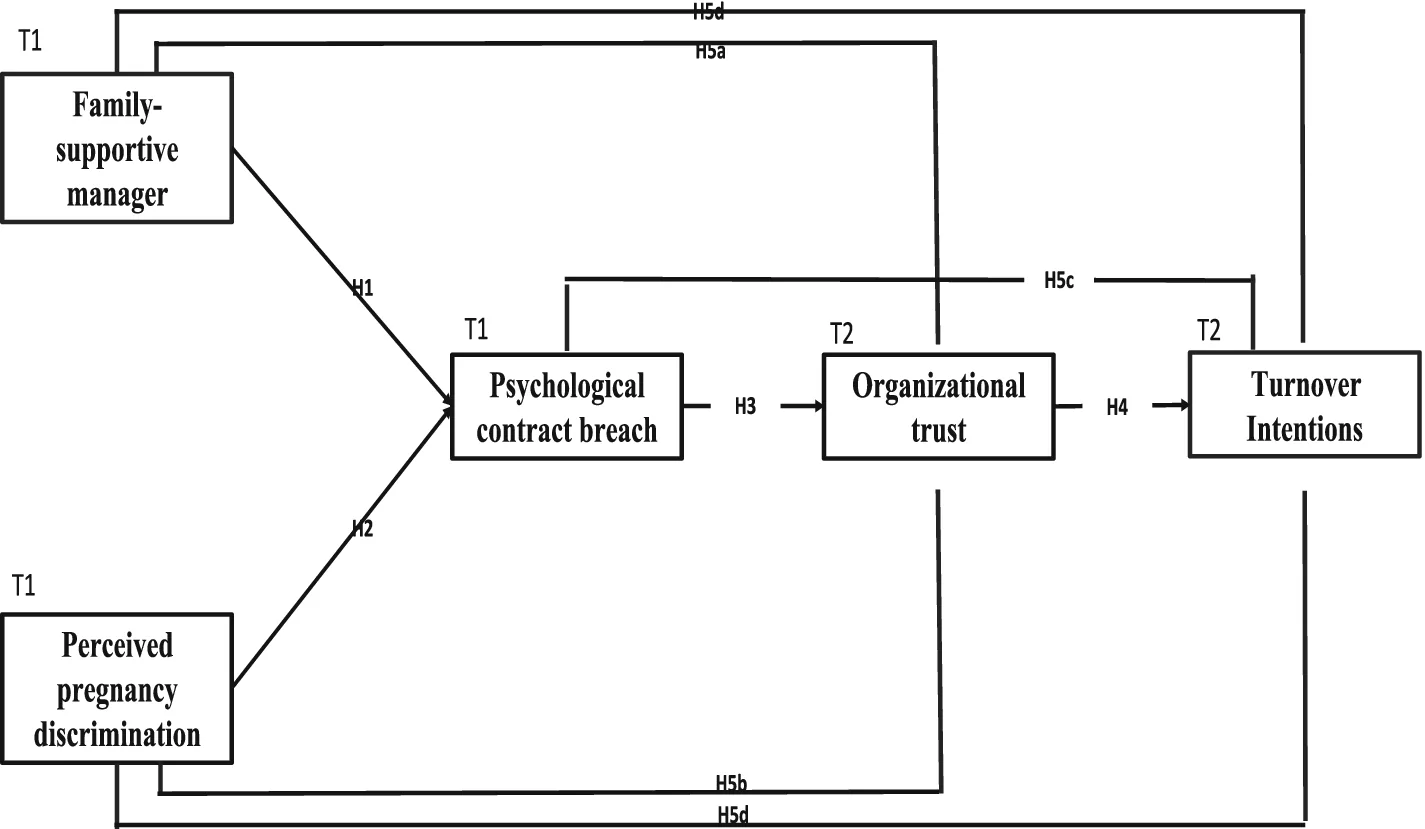
Research model.

Finally, remote work may reflect a shift in the relationship between organizations and pregnant and post-maternity leave employees, providing a new angle from which to examine it. More specifically, we propose to test the direct effect of family-supportive manager and perceived pregnancy discrimination on psychological contract breach. To the best of our knowledge, no previous research has analyzed these relationships, although a correlation has been found between manager support and psychological contract breach (e.g., Agarwal and Bhargava, 2014; López Bohle et al., 2017) and between perceived discrimination and psychological contract (e.g., DelCampo and Blancero, 2008; Goltz, 2010).

Furthermore, changes in boundary characteristics may not always be accompanied by changes in organizational norms and attitudes (Clark, 2000). Family caregiving was found to lead to turnover intentions among women (Ahmed and Riaz, 2025). Yet, the relationship between pregnancy discrimination and turnover intentions has not received adequate attention in the literature (Hackney, 2017). More generally, there is a gap in research exploring how family-supportive manager and perceived pregnancy discrimination affect turnover intentions among pregnant and post-maternity employees in the context of remote work.

## 2 Theoretical background

### 2.1 Theoretical framework

This study synchronized two theories, that of gendered organizations and that of work-family boundary, to explain the phenomenon under investigation. According to Acker (1990, pp. 139–147), organizations are ‘gendered’ meaning that “advantage and disadvantage, exploitation and control, action and emotion, and meaning and identity are patterned through and in terms of a distinction between male and female, masculine and feminine.”

Organizational logic is considered to be a central process that determines how organizational policies and practices are taken for granted and perceived as legitimate, although it reproduces a gendered organization (Acker, 1990). Viewing the organization as a process rather than a relatively static entity (Acker, 1998) leads to a different interpretation of organizational logic in the new economy (Williams et al., 2012). To effectively use this theory, it is important to be aware of the organizational context (Britton, 2000). Acker (1998) argued that workplace flexibility may alter the boundaries between family and work. In the current age, remote and hybrid working are gaining popularity, maintaining work-family boundaries has become increasingly important for employees. Therefore, the present study drew also on the work-family boundary theory.

The basic premise of Clark's (2000) work-family boundary theory is that work and family affect each other so that neither employers nor individuals can ignore one without potentially jeopardizing the other. Although the cultures, means, and goals of the work and home domains are usually different, individuals often manage to integrate the two worlds to some degree (Ashforth et al., 2000). There is no single desirable state of integration or segmentation (Clark, 2000). Boundaries are characterized by psychological and physical permeability, allowing elements from one domain to enter the other (Clark, 2000). For example, individuals who identify with a particular domain tend to exhibit greater commitment and contribute more to that domain (Barhate and Hirudayaraj, 2021).

### 2.2 Family-supportive manager and psychological contract breach/violation

Family-supportive manager is defined as “the extent to which managers [are] supportive and sensitive to employees’ family responsibilities” (Thompson et al., 1999, p. 401). Therefore, management is considered to provide emotional support through greater empathy for personal issues (Yu et al., 2022). According to the work-family boundary theory, a manager with high family-domain awareness recognizes the family commitments and challenges of border crossers (Clark, 2000). Working from home is more beneficial for women than for men in reducing work-home conflict, if it is combined with managerial support (van der Lippe and Lippényi, 2020). Flexible work policies may have limited benefits if managers do not encourage employees to use them but evaluate their work based on their physical presence (Clark, 2001).

Morrison and Robinson (1997, p. 230) made a conceptual distinction between perceived contract breach and violation: *perceived breach* refers to the cognition that one’s organization has failed to meet one or more obligations of one’s psychological contract commensurate with one’s contribution. The term *violation* refers to an emotional and affective state that may, under certain conditions, follow from the belief that one’s organization has failed to adequately fulfill the psychological contract.

Previous studies have shown a negative correlation between manager support and psychological contract breach (e.g., Agarwal and Bhargava, 2014; López Bohle et al., 2017). To the best of our knowledge, research on the relationship between family-supportive manager and psychological contract breach in remote and hybrid work arrangements is lacking. We examine whether perceptions of family-supportive manager lead to lower psychological contract breach in pregnant and post-maternity employees during the remote and hybrid work experience. We posited the following hypothesis:

H1: Family-supportive manager correlates negatively with psychological contract breach and violation.

### 2.3 Perceived pregnancy discrimination and psychological contract breach/violation

Pregnant women may be subject to exaggerated gender stereotypes that lead to their discrimination (Bragger et al., 2002). Studies that have been conducted on pregnant women show that they are perceived negatively (e.g., Hackney, 2017; Masser et al., 2007). Despite laws that aim to protect women from discrimination in the workplace during pregnancy and post-maternity leave, women experience both formal and informal discrimination (Cheung et al., 2022). Pregnant women are aware of their potential vulnerability in the workplace, including demotion and even termination (Hackney, 2017). If organizations lack regulations or family-friendly policies that support female employees with children, employees must negotiate the boundaries of the work-family interface (Clark, 2000).

To the best of our knowledge, no studies have examined the relationship between perceived pregnancy discrimination and psychological contract breach and violation. Nevertheless, other studies have indicated the presence of such a relationship. For example, DelCampo and Blancero (2008) discovered that perceptions of discrimination were inversely related to psychological contract fairness among Hispanic business professionals in the US. Similarly, in a qualitative study Goltz (2010) found that women who experienced discrimination changed their beliefs about fairness at the universities where they worked. Our hypothesis extends Acker’s theory of gendered organizations (1990) by identifying one of the mechanisms that reproduce gender inequality under conditions of remote work. We formulated the following hypothesis:

H2: Pregnancy discrimination correlates positively with psychological contract breach and violation.

### 2.4 Psychological contract breach/violation and organizational trust

Organizational trust is defined as “positive expectations individuals have about the intent and behaviors of multiple organizational members based on organizational roles, relationships, experiences, and interdependencies” (Shockley-Zalabak et al., 2000, p. 37). It encompasses work-based relationships between an employee and the rest of the organization (Huff and Kelley, 2003). The development of trust within an organization depends on the context in which it occurs (Kidron et al., 2016). Organizational trust is a foundational element of successful remote work (Pyöriä, 2011), therefore, working remotely is likely to affect the trusting relationship between managers and employees (Charalampous et al., 2023).

Generally, contracts can have a positive impact on trust by creating an environment that fosters it (Järvinen and Branders, 2020). The emergence of trust is a function of the presence of risk and interdependence between the two sides. But trust also entails vulnerability when it is violated in working relationships (Gillespie, 2012). Work-life interactions, such as remote or hybrid work, must meet certain conditions, including the employee being personally valued (Lambert, 2000), as indicated by studies demonstrating a negative relationship between psychological contract breach and organizational trust (e.g., Kidron and Gedalya, 2026). When employees experience dissatisfaction and an imbalance in reciprocal relationships, their organizational trust decreases (Archimi et al., 2018). We are not aware of research conducted on the relationship between psychological contract breach and organizational trust among women during pregnancy and post-maternity leave. We propose the following hypothesis:

H3: Psychological contract breach and violation correlate negatively with organizational trust.

### 2.5 Organizational trust and turnover intentions

Turnover intentions is defined as the probability that an individual will leave the organization in the near future (Mowday et al., 1984). Employees’ turnover intentions represent their critical commentary on broken organizational promises (De Clercq, 2025). According to a meta-analysis by Armitage and Conner (2001), intentions are solid predictors of behavior. Similarly, turnover intentions are the final cognitive step in the decision-making process leading to voluntary turnover (Fishbein and Ajzen, 1975; Lambert, 2006). In a meta-analysis, Cotton and Tuttle (1986) found that family responsibilities and gender were correlated with turnover intentions. Yet, organizations are interested in understanding and managing the determinants of employee turnover intentions (Agarwal and Bhargava, 2014).

Organizational trust is important in determining attitudes and outcomes of individuals at the workplace (Gellatly and Withey, 2012). A lack of trust is one of the main reasons behind an employee’s intention to leave an organization (Costigan et al., 2013). According to a meta-analysis by Dirks and Ferrin (2002), trust is strongly associated with turnover intentions. This correlation is supported by other studies, like those of female kindergarten teachers conducted in China (Zhao et al., 2022), social workers in South Korea (Cho and Song, 2017), and service employees in the United Arab Emirates (Zeffane and Bani Melhem, 2017). We propose the following hypothesis:

H4: Organizational trust correlates negatively with turnover intentions.

### 2.6 Psychological contract breach/violation and organizational trust as mediators

This study aims to examine the indirect associations between family-supportive managers, perceived pregnancy discrimination, and turnover intentions, via psychological contract breach/violation and organizational trust, among pregnant and post-maternity leave employees working remotely or in a hybrid setting. The transition from on-site to remote or hybrid work has made work-home boundaries more flexible and permeable, blending work and family life (Clark, 2000). The question is whether changes in work-family boundaries are matched by changes in organizational norms and attitudes. Following Acker's (1990) theory, we examine whether the organizational logic is gendered when pregnant and post-maternity women work off-site, by analyzing this contextual dynamic from a female perspective.

Research indicates that managerial and organizational support plays a critical role in employee turnover intentions (e.g., Guo et al., 2024; Hyunkang and Gordon, 2024). Cegarra-Leiva et al. (2012) have shown that a supportive organizational work-life climate is negatively associated with turnover intentions. Kaduk et al. (2019) found that employees who work remotely and perceive their organization as less supportive of family issues have higher turnover intentions. The relationship between pregnancy discrimination and turnover intentions has received limited attention (Hackney, 2017). In a meta-analysis, Hyunkang and Gordon (2024) found that perceived organizational fairness led to lower turnover intentions. Other studies have shown that gender discrimination can predict turnover intentions (e.g., Velez et al., 2018; Yong Kim et al., 2017). We aim to provide new insights by examining the indirect relationships of family-supportive manager and perceived pregnancy discrimination with turnover intentions through two mediators: psychological contract breach and organizational trust.

First, we argue that psychological contract breach will mediate the relationship between family-supportive manager and pregnancy discrimination on the one hand, and organizational trust on the other. Psychological contract breach was found to be related to turnover intentions among nurses (e.g., Yin et al., 2026). Huffman et al. (2022) found that psychological contract breach mediated the relationship between distributive justice and work overload experienced by employees returning from furlough after cutbacks during the COVID-19 pandemic. Additionally, a breach of psychological contract mediated the interaction between job insecurity and employee stress (Shin, 2019) and between bullying and job satisfaction (Kakarika et al., 2017).

Second, we suggest that organizational trust mediates the relationship between psychological contract breach and turnover intentions. A study on psychological contracts found that trust played a mediating role because of the harm caused by breached employer commitments (Hornung and Glaser, 2010). Trust was found as a mediator between psychological contract breach and turnover intentions (Clinton and Guest, 2014). Agarwal and Bhargava (2014) also found that trust mediated the relationship between psychological contract breach and turnover intentions, as well as affective commitment.

Finally, although previous studies have examined psychological contract breach and organizational trust as separate explanatory factors, little attention has been given to their joint impact on women’s family-related workplace experiences and turnover intentions. To date, these mediating factors have mostly been examined separately. We propose this pathway because family supportive manager and perceived pregnancy discrimination represent salient workplace experiences through which women evaluate the organization’s treatment of them during pregnancy and post-maternity leave.

Psychological contract breach and organizational trust represent conceptually distinct but related constructs. Limited managerial support and/or pregnancy-related discrimination may be perceived as inconsistent with expectations of fair and supportive treatment, thereby contributing to perceptions of psychological contract breach. Psychological contract breach is an assessment of how well an employer fulfills promises, which can affect employee attitudes, such as trust. Based on this, we put forward the idea of an indirect pathway that links a family-supportive manager and perceived pregnancy discrimination to turnover intentions, via psychological contract breach and organizational trust. Based on the above discussion, we proposed the following hypotheses:

H5a: Psychological contract breach and violation mediate the relationship between family-supportive manager and organizational trust.

H5b: Psychological contract breach and violation mediate the relationship between pregnancy discrimination and organizational trust.

H5c: Organizational trust mediates the relationship between psychological contract breach and violation and turnover intentions.

H5d: Psychological contract breach/violation and organizational trust mediate the relationship between family-supportive manager and pregnancy discrimination on one hand and turnover intentions on the other.

## 3 Methodology

### 3.1 Procedure, sample, and participants

Survey data were collected via iPanel,^1^ a large, representative online database that adheres to the highest standards set by ESOMAR. iPanel has previously demonstrated reliability in academic studies (e.g., Kidron and Vinarski-Peretz, 2024). The recruitment and maintenance of probability-based online panels is subject to rigorous controls and has recently established itself as an important survey mode (Couper, 2017). Data were collected in May–June 2023, with a three-week interval between the two waves.

The participant recruitment process included the following steps: First, iPanel recruited salaried women aged 20–50 from across Israel. Geographical quotas were applied in proportion to the number of employed women in each of the country’s five regions. Second, respondents’ eligibility was determined based on their responses to three filter questions: (1) whether they were salaried employees who had returned from maternity leave within the preceding year and were currently working; (2) whether they were salaried employees who were pregnant and currently working and, if so, whether their employer was aware of the pregnancy; and (3) whether their organization permitted them to work remotely. Respondents had to satisfy either the first or second criterion and confirm that their organization permitted remote working.

Participants’ responses from the two surveys were matched using a unique identification code assigned to each panel member by iPanel. This procedure preserved participants’ anonymity, as researchers had no access to any personally identifiable information. Participants received points redeemable through a gift card in return for completing the survey. The panel provider also incorporated attention-check questions to identify and exclude respondents who provided inattentive or automated responses.

In the first wave, 508 women, either pregnant or on post-maternity leave, responded. In the second wave, 438 women participated. A total of 406 women participated in both waves and formed the sample of the study. To assess potential attrition bias, we compared the demographic characteristics measured at Time 1 of participants who completed both waves (*n* = 406) with those who completed only Time 1 (*n* = 102). Independent-samples t-tests were conducted for continuous variables (age and seniority), while chi-square tests were used for categorical variables (marital status, education and organizational level). No statistically significant differences were found between the two groups, suggesting that attrition was not systematically associated with the characteristics examined.

70 respondents (17.2%) worked entirely remotely (with occasional in-office meetings as necessary), 184 (45.3%) worked remotely for a fixed number of days per week, and 152 (37.4%) worked remotely upon their request. Regarding the remote work policies of the respondents’ organizations: 38 (9.4%) reported that the organization allowed employees to work entirely remotely; 197 (48.5%) reported that the organization allowed remote work a fixed number of days per week; and 171 (42.1%) reported that the organization allowed remote work upon request. The age of the respondents ranged from 20 to 48 years: 30.5% were aged 20–29, 34.7% were aged 30–34, and 34.7% were aged 35–48. Regarding tenure in the organization, 18.2% of employees had up to 1 year of experience, 41.1% had 2–4 years of experience, and 40.6% had 5 or more years of experience. Of the participants, 28.1% had a high school education and 71.9% had an academic degree; 65% were at the employee level and 35% at the managerial level; 31% worked in the public sector and 69% in the private sector.

### 3.2 Measures

The scales used in this study were derived from standardized instruments whose reliability has been previously validated. To ensure the reliability of the scales, we conducted additional validity and reliability analyses. The scales, which originated in English, were translated into Hebrew using the back-translation method. All the items were scored on a Likert scale, ranging from 1 (Strongly disagree or not at all) to 5 (Strongly agree or to a great extent). The respondents were asked to evaluate each item as concerning pregnancy or returning to the organization after maternity leave. To reduce the potential influence of common method bias (CMB), particularly between the two mediator variables, survey data were collected at two different chronological points (T1/T2) (Podsakoff et al., 2024). In addition, CMB was tested using the common latent factor (CLF) methods, namely, an additional latent factor uncorrelated with the five substantive factors and equal loadings on all other items. This did not improve model fit (Δ*χ*^2^ = 0.05, Δdf = 1, *p* = 0.818), and its loading was non-significant (*p* = 0.640). A single-factor model (Harman’s test) fitted poorly (CFI = 0.664, RMSEA = 0.153) and significantly worse than the five-factor model. Common method bias is therefore unlikely to account for the observed relationships.

*Family-supportive manager* (T1) was based on the scale developed by Thompson et al. (1999). The scale includes three items (e.g., “My manager is quite accommodating of family-related needs”). Cronbach’s alpha was 0.88.

*Perceived pregnancy discrimination* (T1) was measured using Hackney's (2017) scale, which includes nine items (e.g., “At work, pregnant employees receive fewer opportunities”). Respondents estimated the extent to which they felt discriminated against because of pregnancy in the past or present. Cronbach’s alpha was 0.92.

*Psychological contract breach and violation* (T1) was measured using a global scale developed by Robinson and Wolfe Morrison (2000). The scale includes nine items, divided into two dimensions: perceived breach of the psychological contract, which includes five items (e.g., “Almost all the promises made by my employer during recruitment have been kept so far”), and feelings of violation, which includes four items (e.g., “I feel that my organization has violated the contract between us”). To assess the discriminant validity of the two dimensions of the scale, we conducted a confirmatory factor analysis (CFA). The one factor breach scale was assessed against the original two dimensions (breach, violation). Parallel analysis retained one factor (first eigenvalue was 6.09, 68% shared variance across all items). In the full measurement model, the two dimensions correlated highly 0.87 (95% CI [0.82, 0.91]), above the proposed 0.85 benchmark for discriminant validity (Brown, 2015), and perceived breach failed the Fornell-Larcker criterion, as its AVE (0.56) was lower than the variance it shared with violation (*r*^2^ = 0.75; Fornell and Larcker, 1981). Moreover, cross-dimension item correlations were substantial (mean *r* = 0.57, against 0.66 and 0.81 within dimensions; HTMT = 0.79; Henseler et al., 2015), and both dimensions related to manager support, pregnancy discrimination, trust and turnover intention in the same direction, with differences of 0.08 at most. Psychological contract breach was, therefore, retained as a single factor.

Consistent with the sense-making literature (Weick, 1995), Parzefall and Coyle-Shapiro (2011) found that employees’ emotional, attitudinal, and behavioral responses to contract breach are intertwined. Therefore, this measure reflects the overall negative experience of the psychological contract, rather than the two dimensions independently. Researchers have also used all nine items in a single measure in other studies (e.g., Botha and Steyn, 2023; Mensah et al., 2024). Additionally, Morrison and Robinson (1997) have argued that there is a difference between contract breaches and violations, “although, in reality, the judgments may be intertwined” (p. 243). Cronbach’s alpha was 0.93.

*Organizational trust* (T2) was measured using a global questionnaire developed by Huff and Kelley (2003). The scale includes four items (e.g., “My organization has a high level of trust”). Cronbach’s alpha was 0.91.

*Turnover intentions* (T2) were measured using a universal scale adapted from the work of Sjöberg and Sverke (2000). The scale consists of three items (e.g., “I am actively looking for another job”). Cronbach’s alpha was 0.82.

*Control variables* included socio-demographic characteristics and organizational aspects. At the individual level, the variables were employee seniority (years) and rank in the organization (employee vs. manager). These socio-demographic characteristics have been found to be associated with turnover intentions (Lambert, 2006). At the organizational level, we added the two variables: gender dominance in the organization (female majority, male majority, gender equality) and remote work policy for employees (fully remote work, remote work a fixed number of days per week, and remote work at the employee’s request). Workplace policy was found to be related to turnover intentions (Krøtel and Villadsen, 2016).

### 3.3 Research ethics

Ethical approval for the study was granted by the Institutional Ethics Committee of the Max Stern Yezreel Valley College (Approval No. 2023-45 YVC EMEK). Data were collected via iPanel, which operates in accordance with the ESOMAR ethical guidelines and maintains the confidentiality of its panel members, assigning participants unique numerical identification codes. We only received de-identified data and had no access to personally identifying information. Before beginning the survey, participants were informed of the study’s purpose, the voluntary nature of their participation, the confidentiality of their responses, and their right to discontinue the survey at any time. Participants were only permitted to proceed to the questionnaire after actively providing informed consent.

### 3.4 Analysis strategy

All analyses were conducted using the Mplus V.8.3 statistical package (Muthén and Muthén, 2018). First, we developed a preliminary measurement model to validate the theoretical constructs. To this end, we tested the theoretical construct structures before the final modeling within a measurement model. We calculated internal consistencies using Cronbach’s alpha and McDonald’s omega. Second, to test the research hypotheses, we built a structural equation modeling (SEM) framework that followed the theoretical hypotheses. This modeling approach allows the assessment of both direct and indirect effects.

## 4 Results

We created a measurement model to validate the research constructs. The model fit quality, i.e., goodness-of-fit, met the required thresholds for acceptance (*χ*^2^ = 665.44, df = 306, *p* < 0.001; CFI = 0.960, TLI = 0.954; RMSEA = 0.054, 95%CI [0.048,0.059]; SRMR = 0.054). These results were achieved after the item “There is no pregnancy discrimination in my present job” was removed from the pregnancy discrimination scale because its standardized factor loading was below the recommended 0.40 threshold (*L* < 0.40; Byrne, 2012). This was the only reverse-worded item in the scale, which may have contributed to its poor loading.

Table 1 shows correlations between research variables in two forms: (a) Pearson’s correlation coefficients between indicators and (b) correlations estimated within the measurement model between latent factors. These correlations exceeded 0.60 only for the perceived pregnancy discrimination and psychological contract breach (*r* = 0.66, *p* < 0.001) variables. Thus, although correlations were high, these values remained within the <<0.85, as suggested by Brown (2015). The two forms produced relatively similar correlation coefficients. Factors internal consistencies were assessed by Cronbach’s alpha and MacDonald’s omega and were found to be good (>0.75) to excellent (>0.80). Shared variance percentages were based on the maximum likelihood extraction method and indicated a unique dimension for each factor. Reductions in CFI of the metric and scalar models with respect to the configural model were small (metric vs. configural: ΔCFI = 0.004, RMSEA = 0.001; scalar vs. configural: ΔCFI = 0.004, ΔRMSEA = 0.000). As some differences were related to education, we also tested for invariance across education levels (i.e., high school vs. graduate degree) and found small reductions in model fit indices (metric vs. configural: ΔCFI = 0.002, ΔRMSEA = 0.001; scalar vs. configural: ΔCFI = 0.005, ΔRMSEA = 0.001). These comparisons provided additional validation of the final factor structures, i.e., female employees from different subpopulations perceived the factors similarly, mainly through means of factor loadings.

**Table 1:** Descriptive statistics and correlations between research variables.

Variables	1	2	3	4	5
Family-supportive management	–	−0.46***	−0.40***	0.47***	−0.33***
Perceived pregnancy discrimination	−0.45***	–	0.66***	−0.32***	0.30***
Psychological contract breach	−0.42***	0.57***	–	−0.48	0.51***
Organizational trust	0.42***	−0.31***	−0.49***	–	−0.51***
Turnover intention	−0.26***	0.23***	0.45***	−0.43***	–
Reliability and shared variance					
Percent shared variance	72.04	63.50	62.81	72.79	61.52
Alpha cronbach	0.881	0.925	0.938	0.913	0.824
Omega McDonald	0.882	0.925	0.938	0.915	0.825
Mean	3.80	1.64	1.93	3.75	2.27
SD	0.87	0.72	0.83	0.86	1.05

***p < 0.001. Upper triangular for the measurement model correlations.

### 4.1 Testing of hypotheses

Correlations between variables lend support to the direct hypotheses H1-H4 (Table 2). Family-supportive manager was found to negatively associate with contract breach (*β* = −0.20, *p* < 0.001) as proposed by H1, whereas pregnancy discrimination was positively associated with psychological contract breach (*β* = 0.48, *p* < 0.001) as stated in H2. Psychological contract breach was found to be negatively associated with organizational trust (*β* = −0.40, *p* < 0.001), which provided support to H3. Organizational trust was found to negatively associate with turnover intentions (*β* = −0.28, *p* < 0.001), an empirical support of H4.

**Table 2:** Indirect effects, standardized coefficients.

Indirect paths	IV → M1	M1 → M2	M → DV	Indirect	95% CI indirect	Direct	Total
Family-supportive manager → Psychological contract breach → Organizational trust	−0.20*** (0.05)	–	−0.40*** (0.06)	0.08* (0.02)	[0.04, 0.13]	0.27*** (0.05)	0.35*** (0.05)
Perceived Pregnancy discrimination → Psychological contract breach → Organizational trust	0.48*** (0.06)	–	−0.40*** (0.06)	−0.19*** (0.04)	[−0.28, −0.12]	0.04 (0.06)	−0.15** (0.06)
Family-supportive manager→ Psychological contract breach → Turnover intention	−20*** (0.06)	–	0.33*** (0.06)	−0.07** (0.02)	[−0.11, −0.03]	−0.05 (0.06)	−0.21** (0.06)
Family-supportive manager→ Organizational trust → Turnover intention	0.27*** (0.05)	–	−0.28*** (0.06)	−0.08** (0.02)	[−0.13, −0.04]	−0.05 (0.06)	−0.21** (0.06)
Family-supportive manager → Psychological contract breach → Organizational trust → Turnover intention	−20*** (0.05)	−0.40*** (0.06)	−0.28*** (0.06)	−0.02** (0.008)	[−0.04, −0.01]	−0.05 (0.06)	−0.21** (0.06)
Perceived Pregnancy discrimination → Psychological contract breach → Turnover intention	0.48*** (0.06)	–	0.33*** (0.06)	0.16*** (0.04)	[0.10, 0.25]	−0.07 (0.06)	0.13* (0.06)
Perceived Pregnancy discrimination → Psychological contract breach → Organizational trust → Turnover intention	0.48*** (0.06)	−0.40*** (0.06)	−0.28*** (0.06)	0.05** (0.02)	[0.03, 0.09]	−0.07 (0.06)	0.13* (0.06)
Psychological contract breach → Organizational trust → Turnover intention	−0.40*** (0.06)	–	−0.28*** (0.06)	0.11*** (0.03)	[0.06, 0.17]	0.33*** (0.06)	0.44*** (0.06)

*p < 0.05, **p < 0.01, ***p < 0.001. Standard errors in parentheses. In squared brackets 95% confidence intervals.

To complete the testing of hypotheses, we examined the hypothesized indirect paths, that is, the combined path from an independent variable to an outcome through the mediating variables. The indirect effect, with a 95% confidence interval, is the result of repeated resampling based on the bootstrapping technique (n_repeats_ = 1,000). As stated in H5a, the results show that psychological contract breach mediated the relationship between family-supportive manager and organizational trust (indirect = 0.08, *p* < 0.05). Similarly, psychological contract breach mediated the relationship between perceived pregnancy discrimination and organizational trust (indirect = −0.19, *p* < 0.001), which lends support to H5b. These two indirect effects were complete. We found support for H5c, so that organizational trust mediated the effect of psychological contract breach on turnover intentions (indirect = 0.11, *p* < 0.01), although the mediation was complementary to the direct effect rather than complete. We also found a complete indirect effect of family-supportive manager on turnover intentions through the combined mediation of psychological contract breach and organizational trust (indirect = −0.07, *p* < 0.01; indirect = −0.08, *p* < 0.01; indirect = −0.02, *p* < 0.01; respectively) and of perceived pregnancy discrimination on turnover intentions through the combined mediation of psychological contract breach and organizational trust (indirect = −0.05, *p* < 0.01; indirect = 0.16, *p* < 0.001, respectively), confirming hypothesis H5d (Table 2). In the model, remote work policy was the only control variable significantly associated with turnover intentions. Employees who worked remotely at their own request reported lower intentions than those who worked fully remotely (*β* = −0.14, *p* < 0.05; Figure 2).

**Figure 2:**
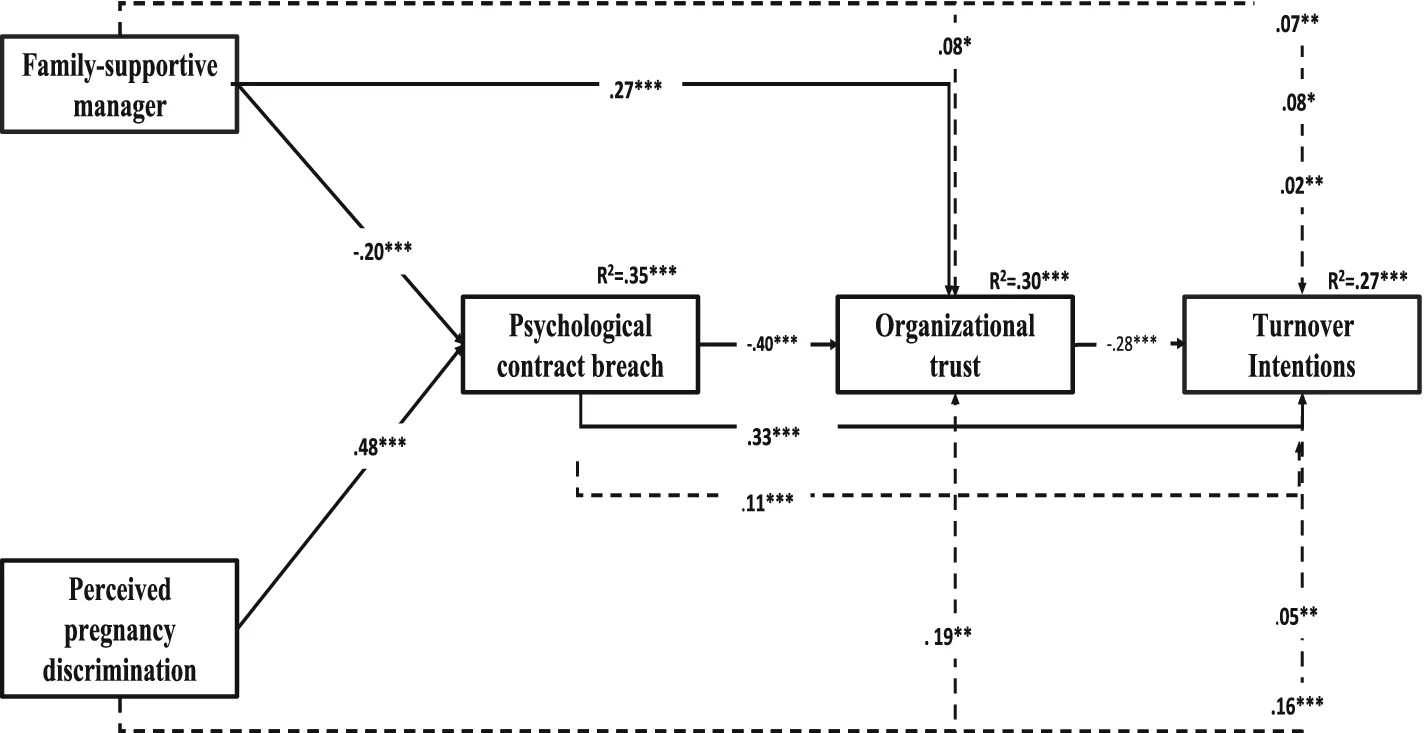
Graphical illustration of the structural model. *p < 0.05 **p < 0.01 ***p < 0.001; Dotted lines indicate indirect paths.

## 5 Discussion

The main purpose of this study was to examine the factors influencing the turnover intentions of pregnant and post-maternity leave employees under remote and hybrid work arrangements. Consistent with our results, this paper makes four main contributions to the body of knowledge.

First, this study contributes to the literature through a micro-level analysis of the theoretical underpinnings of theories of gendered organizations (Acker, 1990) and work-family boundary (Clark, 2000). It reflects the experiences of women under the new working arrangements that have emerged since the onset of the pandemic, shedding light on the new organizational logic. Acker (1990) identified organizational logic as a source of gender inequality, albeit an invisible one. Put differently, although gendered processes at the individual level are often more flexible, they do not follow from or generate gendered processes at other organizational levels (Britton, 2000). This implies that organizations are adopting new work patterns that allow more flexibility and continuity in work and careers, particularly for women, but the organizational logic may not be fully aligned with these new employment patterns, thereby undermining women’s trust in the organization. By incorporating a gender perspective into the analysis (Acker, 1998), we can critically assess and capture how the shift to remote work affects gender dynamics in work organizations.

Second, the findings provide a novel perspective on the turnover intentions of pregnant and post-maternity leave employees. Previous research has paid relatively little attention to the connection between family-supportive manager and perceived pregnancy discrimination on one hand, and turnover intentions on the other (e.g., Cegarra-Leiva et al., 2012; Hackney, 2017; Kaduk et al., 2019). The study’s main contribution is identifying a meaningful pattern of relationships between family-supportive manager, perceived pregnancy discrimination, and turnover intentions. The significant indirect relationships were consistent with the proposed mechanism involving the negative experience of the psychological contract and organizational trust. However, as the two-wave measurement design did not temporally separate all the variables in the proposed model, it does not allow us to determine their temporal ordering or developmental sequence. Accordingly, the findings should be interpreted as providing evidence that is consistent with the proposed relational mechanism, rather than as demonstrating a temporal or developmental sequences. This study is part of recent research (Bates, 2022) that supports Acker's (1998) thesis according to which the processual view is necessary to capture the changing reality. The indirect relationships are consistent with the theoretically proposed ordering in which, as border crossers, women’s perceptions of psychological contract breach are associated with organizational trust, which, in turn, is associated with their turnover intentions. The logic behind these relationships is that “employees do not react to perceived breaches; they react when they perceive a loss of trust” (Agarwal and Bhargava, 2014, p. 1500).

Third, despite the abundance of research on the psychological contract, the present study is the first to examine the effect of the relationship between family-supportive manager and perceived pregnancy discrimination on psychological contract breach. The findings extend the results of previous studies, showing that women who had unsupportive managers or worked in inequitable environments chose to leave their careers (Barhate et al., 2021; Lim and Rasdi, 2019). In general, remote work was more beneficial for women when combined with family-supportive manager (Guo et al., 2024; van der Lippe and Lippényi, 2020), which also led to a lower perceived level of psychological contract breach. As boundary keepers, managers played a crucial role in facilitating border crossers navigating their work-home domains and boundaries (Williams et al., 2012). When managers were more empathetic on work-life issues (Been et al., 2016), women perceived that their psychological contract was not breached.

Finally, this study also offers insights into the positive correlation between perceived pregnancy discrimination and psychological contract breach. Although pregnancy is commonly understood to be a catalyst of discrimination against women, this study reveals its new and significant consequences. Yeung and Shen (2020) identified diversity as a crucial aspect of employees’ psychological contract, in addition to its conventional elements. Masculine norms act as boundaries for women in the workplace (Lim and Rasdi, 2019). Despite the increased acceptance of remote work following the pandemic, the gender stereotypes associated with it have not dissipated (Barhate and Hirudayaraj, 2021). The adoption of remote or hybrid work arrangements does not always result in the requisite changes to organizational culture and norms that would facilitate the utilization of these options by pregnant and post-maternity employees (Clark, 2000), thereby enabling them to develop their careers. The context of remote working for pregnant women and those returning from maternity leave may reinforce perceptions of pregnancy discrimination and related stereotypes, resulting in a perceived breach of the psychological contract.

### 5.1 Limitations and directions for further research

As with any study, this study has several limitations. First, data were collected from pregnant and post-maternity leave employees at two points in time only. The self-reported data are susceptible to common method bias. Although we took steps to minimize such biases, future research should collect data from other sources as well, such as managers, to reduce problems associated with common method bias (Podsakoff et al., 2024). Generally, there are relatively few studies that have focused specifically on pregnant workers, and more research is needed to confirm and strengthen these findings.

Second, although the two-wave design provided temporal separation between some of the study variables, it did not establish the complete temporal ordering specified in the theoretical model. Family-supportive manager, perceived pregnancy discrimination, and psychological contract breach were measured concurrently at T1, whereas organizational trust and turnover intentions were measured concurrently at T2. Therefore, the indirect effects should be interpreted as patterns of associations consistent with the proposed model, rather than as evidence of a causal or temporally unfolding process. Future research should use multiple measurement waves to temporally separate the predictors, mediators and outcome. Such longitudinal studies could provide insight into the evolving nature of the relationships examined in this research.

Third, the psychological contract breach and violation was measured as a one-factor model. Since the model fit of the two dimensions was identical, it was deemed appropriate to measure both dimensions together to present an authentic picture. However, it does not enable us to determine whether the reported correlations are primarily due to perceived breach, feelings of violation, or their shared variance. Therefore, we interpret findings involving this measure as reflecting the combined negative psychological contract experience rather than either component independently. Future studies may consider measuring both dimensions or focusing on only one dimension in a given study.

Finally, as the study was conducted in Israel, it should be interpreted within the country’s particular institutional, legal and cultural context. National and cultural differences during pregnancy, as well as the duration and conditions of maternity leave, may influence women’s expectations and perceptions of managerial support and discriminatory treatment. These contextual factors may affect the reported levels of the variables under research and the strength of the relationships between them. Consequently, the findings should not be assumed to be directly generalizable to countries with different employment protections, welfare arrangements, organizational practices or cultural expectations concerning pregnancy and motherhood. Future research should test the proposed model in different national and institutional contexts, as well as using cross-country comparisons to examine whether these contextual factors strengthen or weaken the model’s findings.

### 5.2 Practical implications

This study’s findings have several practical implications. One of the goals of developing a supportive work-family policy is to enable pregnant women and women on post-maternity leave to continue working and developing their careers and not to “opt out.” First, human resource management (HRM) can organize joint workshops for managers and employees to agree on a ‘*Trust remote work charter*’ to increase managers’ trust in the concept of remote work in general, and in remote employees in particular. If a “Trust remote work charter” exists between managers and employees that explicitly outlines expectations and responsibilities, it can significantly influence the fulfillment of employees’ psychological contracts by aligning both parties’ perceptions of obligations and fostering mutual understanding of the employment relationship in a remote or hybrid work arrangement. Implementing family-friendly policies, coupled with appropriate training, can foster trust between employees and managers, creating a solid foundation for successful remote work arrangements. Rather than relying on informal assumptions, managers and employees could revisit these arrangements when work or caregiving needs change. While such an agreement could help to clarify mutual expectations, its effectiveness should be tested.

Second, organizations should implement and overtly communicate a family-friendly policy that supports pregnancy and post-maternity leave employees, allowing women to work remotely or in a hybrid arrangement without affecting their work assignments or career opportunities. Family-friendly policies, such as remote and hybrid work for pregnant and post-maternity leave employees require the implementation of practices that permeate multiple levels within the organization. These policies would allow employees to balance their work responsibilities with their caregiving responsibilities during this stage of their lives. HR departments could provide managers with guidance on consistently responding to these requests and establish a confidential route for raising concerns about pregnancy-related treatment. These steps are particularly relevant given the study’s focus on family-supportive managerial behavior and perceived pregnancy discrimination.

Third, policy availability alone may not be sufficient if employees feel unable to use flexible working arrangements. HR departments should periodically review whether access to flexibility and development opportunities is applied consistently for pregnant and post-maternity employees. Concurrently, managers could discuss work goals, workload and schedule with pregnant and post-maternity employees. These practices may help to create clearer psychological contracts and build more trustworthy employment relationships.

Finally, at the organizational level, a supportive work-family culture can create organizational norms that respect employees’ family time and facilitate the use of flexible work options (van der Lippe and Lippényi, 2020). Moreover, at the managerial level, a family-supportive organizational culture has the potential to influence managerial attitudes and norms by fostering an environment that values work-life balance and accommodates employees’ family responsibilities. Such a culture could mitigate discrimination against pregnant women and women on post-maternity leave, as their needs and obligations outside of work are better understood and accepted within organizational norms.

## 6 Conclusion

This study reveals insights into pregnant and post-maternity leave employees’ intention to leave under remote and hybrid work arrangements. The findings of this study exemplify Acker's (2006) concept of inequality regimes, which refers to loosely interrelated practices, processes, and policies that result in gender inequalities, specifically for pregnant employees and those on post-maternity leave. The study provides preliminary evidence on the attitudes of pregnant employees and those on post-maternity leave toward the relationship between family-supportive manager, perceived pregnancy discrimination, and psychological contract breach. We also have demonstrated that psychological contract breach and organizational trust mediated between family-supportive manager and perceived pregnancy discrimination on turnover intentions. Practically, although organizations can change the boundaries of the work-home domains (Clark, 2000), this study suggests that family-friendly policies may be effective if, in parallel, organizations redefine their work relations to create beneficial and supportive work environments for employees who are pregnant or on post-maternity leave.

## Data Availability

The raw data supporting the conclusions of this article will be made available by the authors, without undue reservation.

## Footnotes

1
www.ipanel.co.il
